# Elevated interleukin-6 levels predict short-term flare in systemic lupus erythematosus

**DOI:** 10.3389/fimmu.2026.1825342

**Published:** 2026-05-08

**Authors:** Borja del Carmelo Gracia Tello, José Miguel García-Bruñén, Luis Corredor, Natalia Allue Fantova, Eva Ma Calvo Beguería, Rubén García-Muñío, Laura Pérez Abad, Jimena Aramburu Llorente, Beatriz Martinez Muriel, Paula Vidales Miguélez, Roxanna Alexandra Morante Mendoza, Julia Martínez Artigot, Noelia Chicano Nieto, Jorge Álvarez Troncoso, Luis Martinez-Lostao, Adela Marín Ballvé

**Affiliations:** 1 Department of Internal Medicine, Hospital Clínico Universitario Lozano Blesa, Zaragoza, Spain; 2 Department of Immunology, Hospital Clínico Universitario Lozano Blesa, Zaragoza, Spain; 3 Pharmacy Department, Hospital General San Jorge, Huesca, Spain; 4 Department of Internal Medicine, Hospital General San Jorge, Huesca, Spain; 5 Department of Internal Medicine, Hospital Universitario Miguel Servet, Zaragoza, Spain; 6 Department of Internal Medicine, Hospital Universitario La Paz, Madrid, Spain

**Keywords:** Systemic lupus erythematosus, Interleukin-6, Predictive biomarker, SLEDAI-2K, Disease activity, Prospective study

## Abstract

**Background:**

Systemic lupus erythematosus (SLE) is characterized by fluctuating activity and unpredictable flares that contribute to cumulative organ damage. Biomarkers capable of identifying patients at risk of near-term disease reactivation are needed. Interleukin-6 (IL-6) has been associated with SLE activity, but its prospective predictive value remains uncertain.

**Methods:**

We conducted a prospective, longitudinal, single-center study (2017–2023) including 188 adult with SLE. Serum IL-6 levels were measured at routine and unscheduled visits. Patients were categorized using a prespecified cutoff of 5 pg/mL. Disease activity was assessed using SLEDAI-2K (with values >4 suggesting active disease) and organ-specific manifestations. Prospective (lagged) associations were analyzed using the log-rank test. Discriminative performance was evaluated using ROC analyses and multivariable models adjusted for clinical confounders. IL-6 was compared with anti–double-stranded DNA antibodies and complement components C3 and C4.

**Results:**

During follow-up, 48% of patients had IL-6 levels >5 pg/mL. IL-6 >5 pg/mL was associated with a shorter time to subsequent flare, with a median time to SLEDAI-2K >4 that was 10 months shorter. Elevated IL-6 also predicted arthritis, nephritis, serositis, and hematologic manifestations. Concurrently, IL-6 demonstrated greater discriminative performance than anti-dsDNA, C3, and C4.

**Conclusions:**

Elevated circulating IL-6 levels are associated with short-term disease reactivation in SLE and may complement established biomarkers in longitudinal disease assessment.

## Introduction

Systemic lupus erythematosus (SLE) is a chronic autoimmune disease characterized by varied clinical manifestations involving multiple organ systems. Severity may range from mild cutaneous disorders to life-threatening organ failure. The incidence is highest among young women, who often present with obstetric complications. Disease activity varies over time. In newly-diagnosed patients, disease activity usually decreases after immunosuppressive therapy. However, about 70% of patients exhibit a relapsing/remitting pattern, resulting in fluctuations between periods of low disease activity or remission and flares. Such fluctuations can also be observed in patients with longstanding disease. High disease activity is associated with greater organ damage and poorer long-term outcomes (1). Flares remain unpredictable and can involve different organs, and sustained periods of either remission or lupus low disease activity state (LLDAS) are difficult to achieve for most patients (2).

Anticipating flares would prompt closer monitoring and enable earlier intervention, which could ultimately reduce the risk of organ damage. Numerous studies have assessed the predictive ability of circulating cytokines, immune mediators such as interferons, metabolomic and proteomic biomarkers, and even machine learning–based marker panels. However, although encouraging results have been reported, none of these tools have demonstrated consistent performance across cohorts sufficient to be considered a gold standard, or are widely available for routine clinical use, with many remaining primarily research tools (3–5).

Interleukin-6 (IL-6) is a pleiotropic proinflammatory cytokine involved in key pathways driving autoimmune disease. IL-6 plays a relevant role in SLE pathogenesis by promoting B cell differentiation, autoantibody production, and T follicular helper cell development. These mechanisms have been linked to the variability in organ involvement that characterizes SLE. In the clinical setting, an association between higher IL-6 concentrations and increased disease activity, including renal involvement and flares, has been described (6). However, whether IL-6 levels can predict near-term SLE flares or sustained long-term remission remains to be determined. To address this gap, we recruited a cohort of patients with SLE who attended regular hospital visits for disease monitoring. IL-6 levels were determined at all visits. Patients were prospectively followed; the occurrence of flares, estimated by the Systemic Lupus Erythematosus Disease Activity Index (SLEDAI-2K) and organ-specific manifestations, was documented at each visit, and its association with IL-6 levels measured at the previous visit was assessed.

## Patients and methods

### Study design and patients

We conducted a prospective, longitudinal, single-center, noninterventional study was designed. Between January 2017 and February 2023, patients with SLE attending University Clinic Hospital Lozano Blesa (Zaragoza, Spain) for routine disease monitoring were recruited; patients newly diagnosed during this period were also included. After the baseline visit, physical examinations and laboratory tests were scheduled at 3, 6, 12, 18, and 24 months, and, when applicable, annually thereafter. Unscheduled visits prompted by patient-reported symptoms were also included. Inclusion criteria were age >18 years and a diagnosis of SLE (either established before study entry or at inclusion). Exclusion criteria included concomitant diseases with symptoms overlapping those of SLE and conditions known to increase circulating IL-6 (including acute infection at the time of sampling or within the preceding month, based on clinical suspicion or other active pathologies). **Supplementary Figure 1** shows the study flow diagram.

### Assessments

Circulating IL-6 levels (Human IL-6 ELISA Kit, Diaclone, Besançon, France) were measured at all visits, regardless of the number attended by each patient. At each visit, a complete physical examination was performed to assess SLE-related manifestations (including arthritis, myositis, neuritis, nephritis, skin lupus, aphthous ulcers, serositis, leukopenia and thrombocytopenia) defined according to the EULAR/ACR classification criteria (7), and a panel of laboratory tests was carried out. Anti–double-stranded DNA (anti-dsDNA) antibodies were determined using a chemiluminescent assay (Werfen, Barcelona, Spain; reference range: <35 IU/mL). Antinuclear (ANA) autoantibodies were determined using an immunofluorescence assay (Aesku, Wendelsheim, Germany; reference range: <1/80 dilution). Complement factors C3 and C4 were assessed by nephelometry (Beckman-Coulter, Brea, CA, USA; reference ranges: 79–152 mg/dL and 10–40 mg/dL, respectively). C-reactive protein (CRP) was determined by nephelometry (Beckman-Coulter, Brea, CA, USA; reference range ≤1 mg/dL). Clinical and laboratory information was used to calculate SLEDAI-2K (24 weighted items across 9 organ systems). SLEDAI-2K values >4 were considered to suggest active SLE. Organ-specific analyses were considered exploratory and interpreted in the context of multiple testing. At each visit, blood sampling for biomarker determination and clinical examination were performed on the same day.

### Ability of IL-6 to predict future SLE activity

Prior studies suggest that IL-6 values >5 pg/mL are associated with increased disease activity and inflammatory burden (6). On the other hand, IL-6 values in the range of 5 pg/mL or higher can also be observed in patients in remission or LLDAS (8). For this reason, a threshold of 5 pg/mL was established to test the predictive ability of IL-6. Thus, at each visit, patients were categorized into two groups according to IL-6 values ≤5 pg/mL or >5 pg/mL. SLEDAI-2K and separate SLE manifestations were assessed at each time point. To evaluate the predictive ability of IL-6, time zero was defined as the visit at which IL-6 was measured, and patients were followed until the first subsequent occurrence of the event of interest (SLEDAI-2K >4 or an organ-specific manifestation), last available visit, or end of follow-up (censoring). Kaplan–Meier curves are presented for descriptive visualization; inferential analyses accounted for within-patient correlation as detailed below.

### IL-6 as a predictor of concurrent disease activity

To assess the association between IL-6 and concurrent disease activity, all available study visits were considered. For each visit, disease activity (SLEDAI-2K and organ-specific manifestations) was analyzed together with the IL-6 value determined at the same time point. This analysis evaluated whether IL-6 levels >5 pg/mL were associated with active state (assumed to occur when SLEDAI-2K was >4) at the same visit. Anti-dsDNA, C3 and C4 were also assessed at all visits to compare performance with IL-6.

### Statistical analysis

Quantitative variables are presented as median (IQR) and categorical variables as counts and percentages. Because patients contributed multiple visits, both prospective (lagged) and concurrent visit-level associations were estimated. The log-rank test was used to analyze the influence of lagged IL-6 levels on time-to-event patterns, and Kaplan–Meier curves are presented for visualization purposes. Two-tailed chi-square tests were used to assess the association between IL-6 levels >5 pg/mL and concurrent SLEDAI-2K >4 and/or organ-specific manifestations.

For biomarker dichotomization, anti-dsDNA, C3, C4, and CRP were categorized using clinically meaningful cutoffs (anti-dsDNA <35 vs ≥35 IU/mL; C3 <79 vs ≥79 mg/dL; C4 <10 vs ≥10 mg/dL, CRP ≤1 mg/dL vs >1 mg/dL). Predictive performance for concurrent activity (SLEDAI-2K >4) was summarized with ROC curves and AUC (with 95% confidence intervals [CI]); AUCs were compared using the DeLong method. Finally, in order to assess the independent association of biomarkers with disease activity, we initially performed a univariate analysis to obtain a first estimation of disease activity risk according to whether levels of IL-6 were >5 pg/mL, anti-dsDNA were ≥35 IU/mL, C3 was <79 mg/dL, C4 was <10 mg/dL, or CRP was >1 mg/dL. A multivariable model was subsequently built with the variables that yielded a significant result in the univariate analysis.

### Ethics

The study was approved by the Medical Research Ethics Committee of University Clinic Hospital Lozano Blesa and conducted in accordance with the Declaration of Helsinki. All patients signed the written informed consent, and data were properly anonymized before uploading them to the databases.

## Results

### Patients and disease evolution

The main clinical characteristics at the first study visit, as well as follow-up information, are summarized in **Table 1**. A total of 188 patients were recruited between January 2017 and February 2023; 38 (20.3%) were newly diagnosed with SLE. One hundred sixty-seven patients (88.8%) attended at least two visits. Almost 90% of patients were female. The median (IQR) number of visits per patient was 4 (3–7), and the median follow-up was 22.6 (12.4–32.1) months. Overall, 90 patients (47.9%) had IL-6 levels >5 pg/mL at least once during follow-up. Seventy-seven patients (41.0%) presented with active disease, assumed to occur when SLEDAI-2K was >4, at least at one follow-up visit. Arthritis, observed in 71 patients (37.8%), was the most frequent clinical manifestation during the study period. Fifty-three patients (28.2%) developed multiorgan involvement (defined as ≥2 distinct clinical manifestations). Most patients received maintenance treated with hydroxychloroquine; more potent immunosuppressive agents, including corticosteroids or belimumab, were used during disease flares.

**Table 1:** Characteristics of patients at first visit and prospective evolution of clinical manifestations*.

Features at baseline visit	
Age, years	50 (41-64)
Sex, female, n/N (%)	163/188 (86.7)
Newly-diagnosed patients, n/N (%)	38 (20.3)
IL-6, pg/mL	2.7 (1.8-5.2)
Patients with IL-6 >5 pg/mL, n/N (%)	49/188 (26.1)
Anti-dsDNA autoantibody, IU/mL	15.3 (9.8-48.0)
Complement C3, mg/dL	89.0 (74.0-106.0)
Complement C4, mg/dL	18.1 (12.1-22.6)
C-reactive protein, mg/dL	1.6 (0.8-4.1)
SLEDAI-2K	2 (0-4)
SLEDAI-2K >4, n/N (%)	51/188 (27.1)
SLE symptoms, n/N (%)	
Arthritis	44/188 (23.4)
Myositis	4/188 (2.1)
Neuritis	1/188 (0.5)
Nephritis	6/188 (3.2)
Serositis	4/188 (2.1)
Leukopenia	5/188 (2.7)
Thrombocytopenia	13/188 (6.9)
Skin lupus	26/188 (13.8)
Aphthous ulcers	6/188 (3.2)
Treatment(s) at recruitment, n/N (%)	
Hydroxychloroquine	171 (91.9)
Corticosteroids	80 (43)
Other immunosuppressive therapies	57 (30.6)
Biological treatment	17 (9.1)
Belimumab	16 (8.6)
Prospective clinical evolution throughout the study period	
Total number of visits, n^a^	947
Visits per patient^a^	4 (3-7)
Follow-up per patient, months^b^	22.6 (12.4-32.1)
Patients with IL-6 >5 pg/mL at least at one visit, n/N (%)	90/188 (47.9)
Visits with IL-6 >5 pg/mL, n/N (%)^c^	166/759 (21.9)
Visits with disease activity reported, n/N (%)^c^	
SLEDAI-2K >4	182/759 (24.0)
Arthritis	121/759 (15.9)
Myositis	11/759 (1.4)
Neuritis	7/759 (0.9)
Nephritis	31/759 (4.1)
Serositis	16/759 (2.1)
Leukopenia	22/759 (2.9)
Thrombocytopenia	29/759 (3.8)
Skin lupus	45/759 (5.9)
Aphthous ulcers	18/759 (2.4)

Data are presented as median (IQR) unless otherwise specified. ^a^Including the first visit attended by each patient. ^b^Time elapsed between the first and last visit attended by each patient. ^c^Excluding the first visit attended by each patient.

*IL-6, interleukin-6; SLE, systemic lupus erythematosus; SLEDAI-2K, Systemic Lupus Erythematosus Disease Activity Index.

### Ability of IL-6 to predict disease flare-up

**Figure 1:**
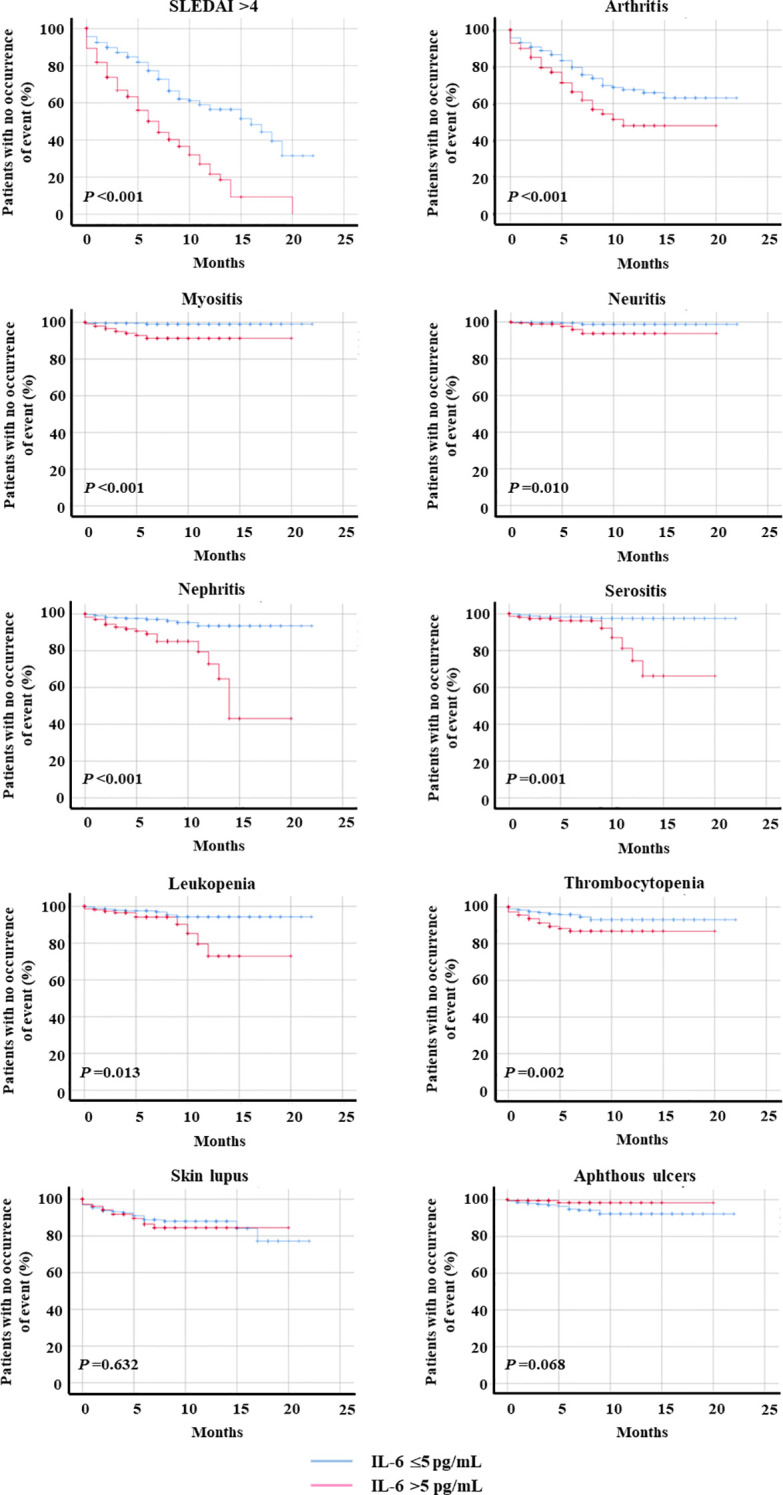
Ability of IL-6 to predict subsequent SLE flare and organ-specific manifestations*. Kaplan–Meier curves are shown for visualization (time zero: the visit at which IL-6 was measured). At each time point, patients were categorized into two groups according to whether they had circulating IL-6 levels ≤5 pg/mL or >5 pg/mL. Outcomes included overall disease activity (assumed to occur when SLEDAI-2K was >4) and individual organ manifestations. Survival curves were generated for the entire study period, and the log-rank test was applied to explore differences in time to flare between the two groups. Data were censored at the date of the last available follow-up. The number of available assessments is indicated for each variable. *IL-6, interleukin-6; SLE, systemic lupus erythematosus; SLEDAI-2K, Systemic Lupus Erythematosus Disease Activity Index.

IL-6 levels >5 pg/mL were associated with a significantly shorter time to onset of SLE clinical manifestations (**Figure 1**), including overall disease flares (top left panel) and organ-specific manifestations such as arthritis, myositis, neuritis, nephritis, serositis, leukopenia, and thrombocytopenia. Time to onset of skin lupus and aphthous ulcers (bottom panels) did not differ significantly between groups. **Table 2** shows the median (IQR) times until the onset of clinical events in each group. Among visits with IL-6 levels >5 pg/mL, the median time to overt flare (assumed to occur when SLEDAI-2K was >4) was 10 months shorter than when IL-6 levels were ≤5 pg/mL. Accordingly, the median time to the onset of SLEDAI-2K>4, nephritis and serositis, was also remarkably shorter in the first group. **Figure 1** shows Kaplan–Meier curves for descriptive visualization; inferential comparisons were performed using the prespecified models accounting for within-patient correlation.

**Table 2:** Time to onset of SLE clinical manifestations according to IL-6 levels*.

Disease activity marker	IL-6 levels at the previous visit		*P* value
	≤ 5 pg/mL	>5 pg/mL	
SLEDAI-2K >4 (n = 862)	16.0 (11.6-20.4)	6.0 (4.6-7.4)	<0.001
Arthritis (n = 875)	16.1 (15.1-17.2)	12.2 (10.5-13.8)	<0.001
Myositis (n = 876)	21.8 (21.6-22.0)	18.5 (17.7-19.4)	<0.001
Neuritis (n = 876)	21.8 (21.6-22.0)	19.1 (18.2-19.9)	0.010
Nephritis (n = 875)	20.9 (20.4-21.6)	14.7 (12.6-16.8)	<0.001
Serositis (n = 876)	21.5 (21.2-21.9)	16.7 (14.7-18.7)	0.001
Leukopenia (n = 876)	20.5 (20.5-21.6)	17.0 (15.2-18.9)	0.013
Thrombocytopenia (n = 876)	20.8 (20.2-21.3)	17.7 (16.9-18.7)	0.002

Results are presented as median (IQR). The months elapsed from the visit at which IL-6 was measured (time zero) to the onset of each clinical manifestation are shown for intervals in which IL-6 was ≤5 pg/mL or >5 pg/mL at the previous visit. Only clinical events with a significant between-group difference in the log-rank test are shown (Kaplan–Meier curves are presented for visualization). The number of available assessments is indicated for each variable.

*IL-6, interleukin-6; IQR, interquartile range; SLE, systemic lupus erythematosus; SLEDAI-2K, Systemic Lupus Erythematosus Disease Activity Index.

### Disease activity at the time of the assessment of circulating IL-6 levels

IL-6 levels >5 pg/mL were strongly associated with overt manifestations of SLE activity at the time of assessment. According to the criteria used to estimate disease activity described above, all 862 study visits with available SLEDAI-2K assessments were categorized as active (SLEDAI-2K >4) or inactive (SLEDAI-2K ≤4). In 102 of the 233 cases (43.8%) in whom SLEDAI-2K was above 4, the simultaneously assessed IL-6 was above 5 pg/mL. In contrast, IL-6 levels >5 pg/mL were observed in only 113 of 629 visits (18.0%) classified as inactive. Median IL-6 levels were approximately twofold higher in active versus inactive visits (**Supplementary Figure 2**). Accordingly, the prevalence of separate clinical manifestations, namely arthritis, myositis, nephritis, neuritis, serositis, leukopenia and thrombocytopenia, was significantly higher in those visits in which the patient had IL-6 levels above 5 pg/mL (**Supplementary Figure 3**).

IL-6 outperformed traditional biomarkers associated with SLE activity, including anti-dsDNA antibodies and complement components C3 and C4. ROC curve analysis demonstrated superior sensitivity–specificity balance for IL-6 in estimating overt disease (SLEDAI-2K >4), with an area under the curve (AUC) of 0.706, compared with the other biomarkers (AUC values of 0.604, 0.569, and 0.613 for anti-dsDNA antibodies, C3, and C4, respectively) (**Supplementary Figure 4**). In multivariable logistic regression models clustered by patient, all biomarkers were independently associated with disease activity; however, IL-6 showed the highest odds ratio: 3.46 (95% CI: 3.01–3.98), compared with 2.10 (1.84–2.40), 1.50 (1.32–1.72), and 2.81 (2.29–3.45) for anti-dsDNA antibodies, C3, and C4, respectively (**Table 3**).

**Table 3:** Logistic regression analysis of biomarkers associated with concurrent SLE activity accounting for repeated visits per patient*.

Variable (n = 947)	OR	95% CI	*P* value
Univariate analysis			
IL-6 >5 pg/mL	4.10	2.86-5.88	<0.001
Anti-dsDNA ≥35 IU/mL	2.19	1.58-3.04	<0.001
C3 <79 mg/dL	1.84	1.32-2.57	<0.001
C4 <10 mg/dL	4.59	2.86-7.38	<0.001
C-reactive protein >1 mg/dL	1.01	0.72-1.42	0.950
Multivariate analysis			
IL-6 >5 pg/mL	3.46	3.01-3.98	<0.001
Anti-dsDNA ≥35 IU/mL	2.10	1.84-2.40	<0.001
C3 <79 mg/dL	1.50	1.32-1.72	<0.001
C4 <10 mg/dL	2.81	2.29-3.45	<0.001

An univariate analysis considering potential biomarkers was initially performed to calculate ORs (95% CI) for active disease (assumed to occur when SLEDAI-2K was >4) when, at the time of SLEDAI-2K assessment, IL-6 levels were >5 pg/mL, anti-dsDNA autoantibodies were ≥35 IU/mL, C3 levels were <79 mg/dL, C4 levels were <10 mg/dL, or C-reactive protein levels were >1 mg/dL. Only those variables whose associated risk was significant in the univariate analysis were included in the multivariable model, which was carried out to calculate independent ORs (95% CI).

*C3, complement C3 protein; C4, complement C4 protein; CI, confidence interval; GEE, generalized estimating equations; IL-6, interleukin-6; OR, odds ratio; SLE, systemic lupus erythematosus; SLEDAI-2K, Systemic Lupus Erythematosus Disease Activity Index.

## Discussion

In this prospective longitudinal study, we demonstrate that elevated circulating IL-6 levels are associated with a significantly shorter time to a subsequent increase in SLE disease activity and organ-specific manifestations. Patients with IL-6 levels >5 pg/mL experienced earlier loss of disease control, supporting the role of IL-6 as a potential short-term predictor of disease activity. Given the clinical consequences of uncontrolled flares, the identification of a simple biomarker capable of stratifying near-term flare risk remains a priority in SLE management. Among the most encouraging approaches, a flare risk index has been proposed based on the comparison of biomarker panels in two cohorts of 46 and 53 patients with SLE, assessed at the quarterly visit immediately preceding a flare versus a non-flare visit, respectively. The panel achieved high sensitivity (97%) and specificity (98%) for predicting imminent SLE flares in longitudinal cohorts. One of the strengths of the study was the large number of analytes tested to build an effective ensemble of biological indicators. The panel, which is currently being evaluated for clinical application, consists of up to 11 biomarkers, including IL-6, thus requiring comprehensive laboratory testing at each visit (9). Another study, although retrospective, suggested that double positivity for anti-dsDNA and anti-Sm autoantibodies at SLE diagnosis increases flare risk (10). Nevertheless, even if confirmed, this approach would not be useful for estimating flare risk in patients with established disease of several months or years’ duration. Other studies have yielded promising results; however, they often rely on analytes that are not widely available or on multi-marker panels requiring high-throughput technologies such as proteomics and/or advanced machine learning approaches (11–14). Others focus on specific SLE clinical manifestations, particularly renal flares (3, 15). Our study supports further investigation into the value of a single, easy-to-measure cytokine to predict subsequent disease activity over the following months. This conclusion is supported by the prospective design and the large number of paired assessments of circulating IL-6 levels and clinical status, with follow-up extending beyond 12 months in many patients. Furthermore, rather than focusing on a single manifestation, we examined the association between elevated IL-6 levels and subsequent occurrence of the major clinical manifestations of SLE. Kaplan–Meier analyses linked clinical status with IL-6 levels measured at the preceding visit, highlighting the potential value of IL-6 in predicting short-term increases in SLE activity and supporting closer monitoring and/or preemptive therapeutic adjustment. Finally, we demonstrated that elevated IL-6 levels are associated with concurrent disease activity. This association has been previously reported (6). Nevertheless, its superior performance compared with other established biomarkers of SLE activity warrants emphasis.

Our study has limitations. There is no full consensus on objective criteria to define the onset of a flare. To address this limitation, we decided, on the one hand, to use a SLEDAI-2K threshold of >4, based on landmark reports indicating that SLEDAI-2K ≤4 represents the upper limit for LLDAS, provided that there is no activity in major organ systems, no hemolytic anemia or gastrointestinal activity, a physician global assessment ≤1, and prednisone ≤7.5 mg/day (2, 16); and, on the other hand, to examine SLE clinical symptoms separately. The findings regarding the predictive ability of IL-6 were consistent across both approaches.

In summary, our findings provide prospective evidence that routine assessment of circulating IL-6 levels during follow-up may help identify patients at increased risk of short-term disease reactivation. Although external validation in independent cohorts is warranted, IL-6 measurement represents a simple and clinically accessible tool that could complement traditional biomarkers in the longitudinal management of SLE.

## Data Availability

The raw data supporting the conclusions of this article will be made available by the authors, without undue reservation.
