# Supplementary material for: Elevated interleukin-6 levels predict short-term flare in systemic lupus erythematosus
Source: Front Immunol. 2026 May 8;17:1825342. doi: 10.3389/fimmu.2026.1825342 (PMC13194535; doi:10.3389/fimmu.2026.1825342)
Supplement: Supplementary file 1 [file Table1.docx]

**
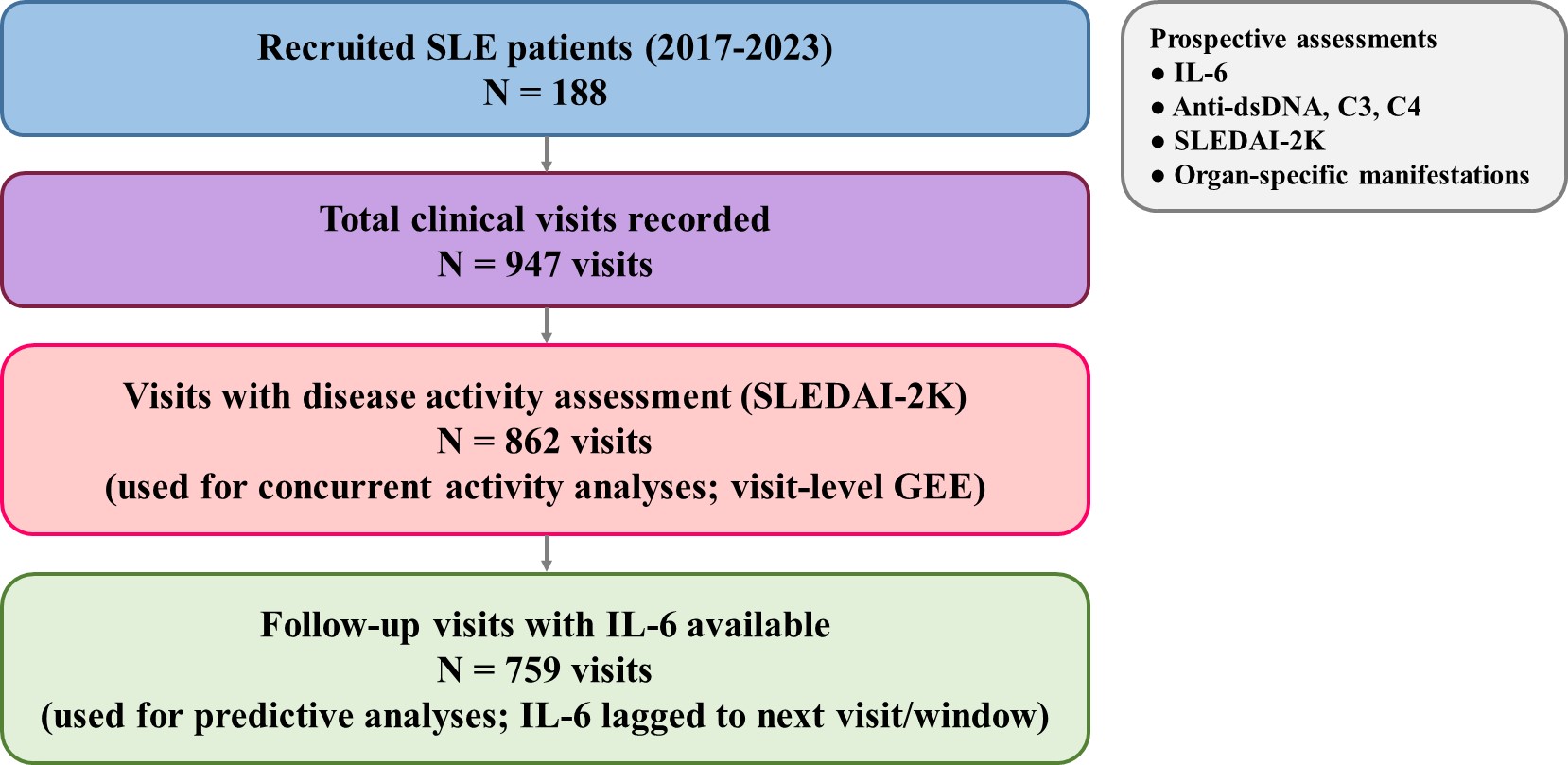
Supplementary Figure S1. Study flow and analytical samples***

SLE patients who attended the University Clinic Hospital Lozano Blesa from January 2017, regardless of the reason (scheduled control, first diagnosis, or flare), were enrolled. After the recruitment visit, scheduled follow-up visits were planned at 3, 6, 12, 18, 24, and 36 months, and every 12 months thereafter until February 2023. Patient-initiated visits for suspected flare were also included. Counts reflect available data for each analysis. Visit-level models account for repeated measures within patients using GEE with robust (sandwich) standard errors (cluster = patient).

*C3, complement C3 protein; C4, complement C4 protein; GEE, generalized estimating equations; IL-6, interleukin-6; SLE, systemic lupus erythematosus; SLEDAI-2K, Systemic Lupus Erythematosus Disease Activity Index.

**
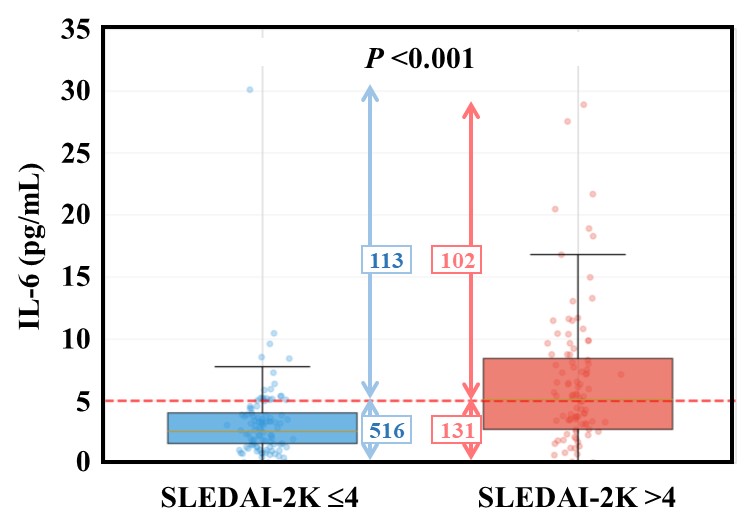
Supplementary Figure S2. Association between SLE activity and IL-6 levels assessed concurrently (visit-level)***

Between-group comparisons and odds ratios were estimated using GEE with robust standard errors clustered by patient. Concurrent assessment of IL-6 levels and disease activity (active disease was assumed to occur when SLEDAI-2K was >4) was available for 862 of 947 visits. The proportion of patients with IL-6 levels >5 pg/mL was compared between activity groups using the two-tailed chi square test. The number of IL-6 determinations above and below the 5 pg/mL cutoff is also shown.

* IL-6, interleukin-6; SLE, systemic lupus erythematosus; SLEDAI, Systemic Lupus Erythematosus Disease Activity Index.

**Supplementary Figure S3. Association between organ-specific SLE manifestations and IL-6 levels assessed concurrently (visit-level)**


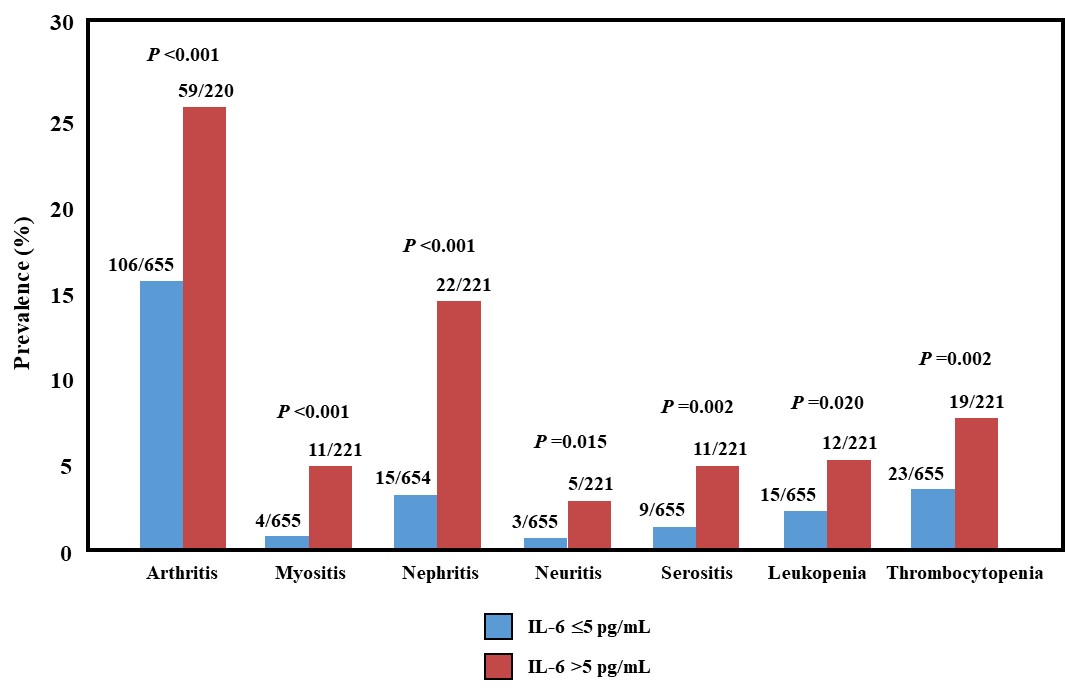


Concurrent assessment of IL-6 levels and organ-specific SLE manifestations was available for 875 (arthritis, nephritis) and 876 (myositis, neuritis, serositis, leukopenia, thrombocytopenia) of 947 visits. Visits were categorized according to IL-6 levels (≤5 vs >5 pg/mL), and the prevalence of each manifestation was compared using the two-tailed chi-square test.

**Supplementary Figure S4. ROC curves evaluating the ability of selected biomarkers to detect concurrent SLE activity***


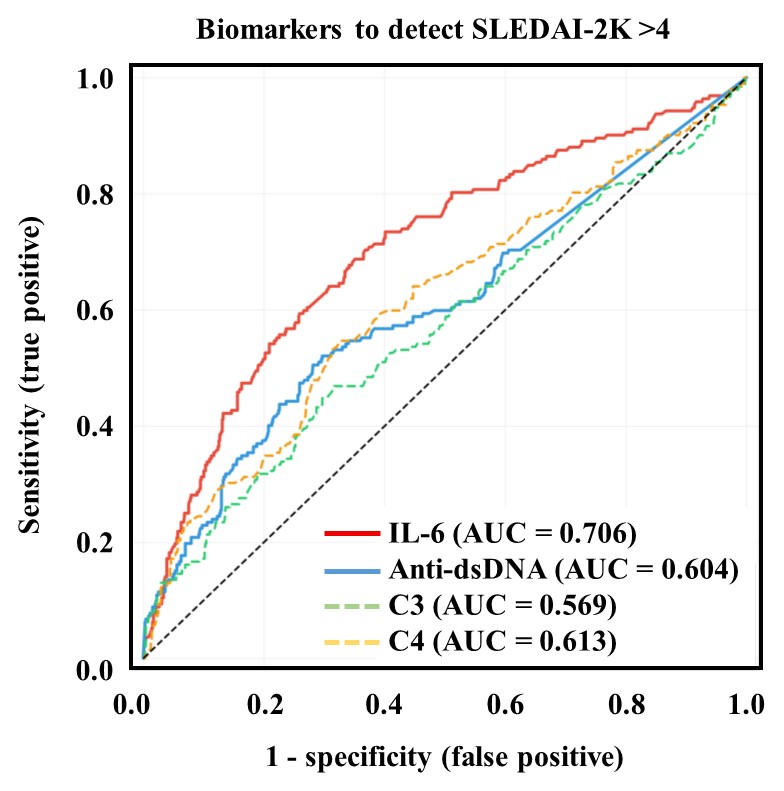


AUCs are reported with 95% CI and compared using the DeLong method. ROC curves assess the ability of the analyzed biomarkers to discriminate patients with active SLE (active disease was assumed to occur when SLEDAI-2K was >4) at the time of assessment. Thresholds defining active disease were: IL-6, >5 pg/mL; anti-dsDNA autoantibodies, ≥35 IU/mL; C3, <79 mg/dL; and C4, <10 mg/dL. The calculations were performed over the 862 visits in which SLEDAI-2K was assessed.

*AUC, area under the curve; C3, complement C3 protein; C4, complement C4 protein; CI, confidence interval; IL-6, interleukin-6; ROC, receiver-operating characteristic; SLE, systemic lupus erythematosus; SLEDAI, Systemic Lupus Erythematosus Disease Activity Index.
